# Estimating Young Children’s Exposure to Food and Beverage Marketing on Mobile Devices

**DOI:** 10.1016/j.cdnut.2024.104505

**Published:** 2024-11-06

**Authors:** Erica L Kenney, Rebecca S Mozaffarian, Jasmine Norris, Frances Fleming-Milici, Sara N Bleich

**Affiliations:** 1 Department of Nutrition, Harvard T.H. Chan School of Public Health, Boston, MA, United States; 2 University of Connecticut Rudd Center for Food Policy and Obesity, Storrs, CT, United States; 3 Department of Health Policy and Management, Harvard T.H. Chan School of Public Health, Boston, MA, United States

**Keywords:** children, pre-teens, preschool-aged, screen time, food and beverage marketing, digital marketing

## Abstract

**Background:**

Food and beverage marketing drives poor diet quality and obesity risk among children. However, it is unknown how much young children are exposed to digital food and beverage marketing on mobile devices like tablets and smartphones.

**Objectives:**

The objective of this study was to estimate how frequently young children, who are particularly vulnerable to advertising, view food and beverage marketing while using mobile devices.

**Methods:**

Seventy-five 2–11-y olds and their parents from Massachusetts participated in this cross-sectional study from 2022 to 2023. Average estimated exposure to food and beverage advertisements and food/beverage brand appearances was calculated for 5 consecutive days using a combination of battery screenshots and average estimates of marketing collected from children’s devices. Generalized estimating equations tested for sociodemographic differences in advertising exposure.

**Results:**

Young children’s estimated exposure to food and beverage advertisements and brand appearances on mobile devices was highly variable, with many children seeing none on a given day but a substantial minority viewing large amounts. Estimated exposure depended on how much a child used either YouTube or a gaming app; there was no exposure on other apps used by children. Higher parental educational attainment was associated with substantially reduced risk of a child viewing 2 or more food or beverage advertisements or brand appearances on a given day (adjusted odds ratio = 0.26, 95% confidence interval: 0.10, 0.70).

**Conclusions:**

Certain children, particularly those from households with lower parental educational attainment, may be at risk for high exposure to digital food and beverage marketing, whereas other children may have minimal risk. Future research should explore exposure in more diverse samples with valid, feasible measures.

## Introduction

As youth mobile device use, including smartphones and tablets, has grown rapidly, even among very young children—time spent on mobile devices among 0–8 y olds increased by over 1000% from 2011 to 2020 [1]—so has concern about potential negative health consequences [[2], [3], [4], [5], [6]]. In addition to widespread concerns about impacts on young people’s mental health, social and emotional skill development, and sleep [[6], [7], [8], [9], [10]], mobile device use also allows for exposure to marketing of health-harming products [11], including foods and beverages that increase the risk of obesity and diet-related chronic diseases [[12], [13], [14]].

Food and beverage marketing [15] has previously been identified through research on television exposure as a determinant of children’s diet quality and risk for childhood obesity [[16], [17], [18], [19], [20], [21], [22], [23]]. Marketing can be particularly influential for younger children, who are forming dietary habits and often cannot understand advertisements’ persuasive intent [20]. Food and beverage marketing is often targeted specifically to children from racial/ethnic minority groups [24,25], and evidence from multiple countries also suggests that children of lower socioeconomic status also have higher exposure [26,27], potentially perpetuating and/or exacerbating racial/ethnic and socioeconomic inequities in diet quality and obesity risk.

In recent years, as youth have moved away from traditional television toward mobile devices, advertising dollars have been shifted toward digital marketing strategies on mobile media platforms [28], including YouTube, streaming video channels, social media, and games. On these platforms, marketing can take different forms. Although advertisements (for example, short videos about a product during a pause in programming, or a still image of a product logo alongside content) are still present, digital marketing also includes brand appearances in social media influencers’ videos [29,30] (for example, a food with branded packaging is conspicuously used as a prop in a video, or an influencer directly promotes a particular product) as well as “liking” or following food and beverage brands on social media [12,29,[31], [32], [33], [34]].

Measuring children’s exposure to digital food and beverage marketing at a person level, however, has proven difficult [35]. Although it was once possible to measure marketing on television with parental reports of their child’s television viewing or by estimating average exposures from market research, both options are now infeasible; parents cannot easily see what children are viewing now that screen time has shifted from shared televisions to personal devices (and given that children’s viewing activities are now so individualized), and market research has been restricted from researchers’ access in most cases [35,36]. Much existing research has focused on identifying the presence of marketing within different child-targeted media itself rather than attempting to measure exposure, finding that child-directed videos are particularly likely to contain appearances of branded food and beverage items (almost all of which are unhealthy), especially on YouTube [29,30,37]. Following a framework for researching children’s digital marketing exposure recently outlined by the WHO [38], several recent studies have documented youth exposure to digital marketing using screen-capture apps, estimating that youth aged 6–19 y are exposed to digital marketing for foods and beverages between 30 and 180 times/wk [[39], [40], [41]]. These studies have been an important start, but, due to the respondent burden related to using screen-capture apps, have only been able to collect data for very short periods of time—for example, Kelly et al. [41] asked youth to screen-record their usage for 2 weekdays and 1 weekend day, but most youth recorded <75% of their usual usage, whereas Nieto et al. [40] asked children to record for only 5 min—running the risk of being unrepresentative of habitual screen media use. Additionally, no studies have yet included young children (that is <6 y old).

Our study builds on prior investigations by combining previously established methods to estimate youth screen device time and marketing exposure to develop objective, quantitative estimates of the average daily exposure to food and beverage marketing on mobile devices of children ages 2–11 y old [42,43]. We also compare these estimates with parent reports of exposure to estimate how valid parental reports are. Lastly, given prior research demonstrating sociodemographic disparities in both screen time and exposure to advertising [24,26,27,44,45], we explore potential sociodemographic correlates of digital food and beverage marketing exposure in this age group.

## Methods

### Study design and sample

This cross-sectional study collected data on 5 d worth of screen usage from a convenience sample of 75 parents of children between the ages of 2 and 11 living in Massachusetts between 2022 and 2023. Parent-child dyads were eligible if the child was between the ages of 2 and 11 y and if the child used a tablet or smartphone. We recruited families by sending flyers with study information to all licensed childcare programs, afterschool programs, and community centers in Massachusetts to optionally distribute to families.

### Measures

#### Sociodemographic characteristics and perceived screen use

After enrollment, parents/caregivers completed a brief survey to report their own and their child’s age, race, ethnicity, gender, and primary language. Parent respondents additionally reported their own educational attainment and total household income and whether they perceived their child as spending too much, too little, or just right amounts of time on their screen devices. Parents also reported if the child used their own device or shared with another family member (including the parent), and whether they used any type of screen-limiting app on their child’s device.

#### Screen use and marketing exposure

**FIGURE 1:**
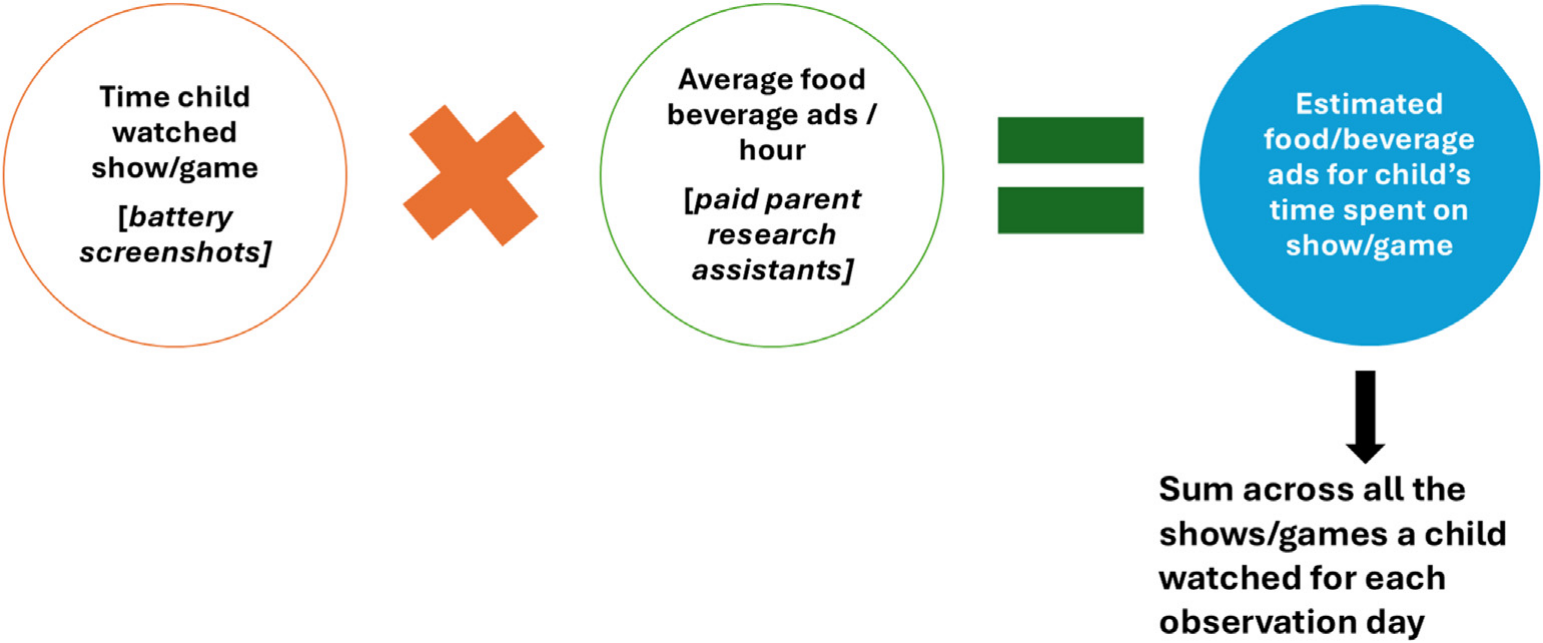
Three-step process for estimating screen use and marketing exposure.

To estimate children’s exposure to food and beverage marketing while using mobile devices, we used a 3-step process, designed to be minimally invasive and feasible for participants (Figure 1).

### Step 1: estimating and characterizing individual children’s mobile device usage

Adapting methods developed by Radesky et al. [42], for 5 consecutive days, participants took daily screenshots of the battery page of the device(s) their child used (hereafter referred to as “device battery screenshots”), which shows how much time a user engaged on each app on their device. Study days included both weekdays and weekend days, depending on when parents started the study. Parents also completed a brief survey at the end of each study day, asking them to specify each app their child had used and then to estimate how much time their child had spent on each app as well as which specific videos or shows their child watched if they used a video-viewing app like YouTube (see Supplementary Instruments 1 and 2 for full survey). These parent-reported data were used to supplement data from the device battery screenshots when it was not possible to identify specific videos on video-viewing apps or when devices were shared. Parents were offered incentives valued at $20 for completing the surveys and $50 for sending the device battery screenshots.

### Step 2: developing standard estimates of average advertisement exposure

From these data, we compiled a list of all games played and specific videos/episodes viewed by children in our study, resulting in 142 unique games and 146 unique videos/episodes. We then recruited parents from the study sample (*n =* 4) to serve as research assistants by watching each video for its full length (or ≤30 min if the video was a movie or longer television episode) and playing each game for 15 min on their own child’s device and documenting what food and beverage ads (including banner, tentpole, and pop-up advertisements using both videos and still images) and brand appearances in the content itself, if any, they saw. We used this approach because the children’s devices were likely to have targeting and advertising cookies in place based on the child’s usage history; parent research assistants using these devices would be more likely to see the types of advertisements typically targeted toward this age group. Parents were trained by study staff to document each food and beverage advertisement or brand appearance they saw while viewing or playing each app using a Google form. Each parent played each app/show on the list for the designated time and tracked the advertisements and brand appearances they saw, giving us a range of estimates for how many ads and what types could be expected to be seen for each app or show. In cases where multiple items appeared in 1 video, each item’s appearance was coded as a separate ad or brand appearance. Parents also took screenshots of the food and beverage advertisements and brand appearances for foods and beverages, allowing us to categorize the types of foods and beverages advertised.

### Step 3: calculating estimated exposure

We then estimated each child’s total food and beverage advertisement and brand appearance exposures for each observation day by multiplying the amount of time the child spent on a given app, show, or game (from the device battery screenshots supplemented with information from the parent daily screen time survey in Step 1) by the average number of food and beverage ads estimated to be shown for that app, show, or game per hour (from Step 2), then summing the total number of ads and brand appearances within each observation day. To validate this approach, we qualitatively compared the estimates derived from this method with estimates derived from a subsample of parents (*n =* 29) who had been invited to record advertisements and brand appearances in real time during Step 1 by sitting next to their children as they played on their devices, watching alongside them, and documenting any advertisements/product appearances seen during that time using a brief form provided by the research team.

### Statistical analysis

After calculating each child’s estimated total screen time and exposure to food and beverage advertisements or branded appearances for each observation day, the distributions of all 3 of these variables were found to be highly left-skewed. We therefore created binary exposure variables based on the data distributions. For food and beverage marketing exposure, we calculated a variable with a value of 1 if the child had seen 2 or more advertisements or brand appearances and 0 if less than that.

Within the advertisement and brand appearances, we used the parent research assistants’ screenshots of the advertisements to categorize the featured foods and beverages as: sugary drinks, sweets (including candy, ice cream, cookies, and other desserts), fast food restaurants, salty snacks, refined grains, cereals/grain products high in added sugars, and all other foods/beverages.

To assess the validity of parental reports of child screen use, we first summed the parents’ estimates of time spent on each app for each child-day from the daily screen time survey (because the survey asked parents to estimate time using ordinal responses, for example 0–15 min, 15–30 min, we used the midpoint of each category for this calculation). Spearman’s correlation coefficients then estimated the correlation between parents’ estimates of the average daily amount of screen time their child viewed and the proportion of observation days on which parents reported their child used various apps with average daily estimates derived from participants’ device battery screenshot data.

In exploratory analyses, we tested whether child age group (categorized as 2–5 y old or 6–11 y old), gender (boys compared with girls and nonbinary), and the parental respondent’s educational attainment (less than college completion, college completion, or graduate degree) were associated with viewing 2 or more food and beverage advertisements or branded product appearances on a given day, adjusting for whether observation days were weekdays or weekend days, using generalized estimating equations (GEE) with logistic regression models that accounted for the clustering of observation days within children. We also calculated a model that additionally accounted for total screen time (in min), to assess whether any differences in estimated marketing exposure were attributable to differences in screen time. Alpha was set at 0.05. All analyses were conducted using SAS 9.4 [46]. Study procedures were approved by the Harvard Chan Institutional Review Board; participating parents gave informed consent and children over 5 provided assent.

## Results

Of the 75 children in the sample, most (*n =* 51, 68%) were 2–5 y old, and there were more boys (*n =* 46, 61%) compared with girls or nonbinary children. Sixty-seven percent identified as non-Hispanic white (*n =* 50) (Table 1). The sample was highly educated, with only 19% of parents having attained less than a college degree. Very few of the children (*n =* 4, 6%) used social media (excluding YouTube). The most frequently used devices by the children in this sample were Apple iPads (*n =* 27, 36%).

**TABLE 1:** Characteristics of convenience sample of Massachusetts parents and young children participating in Kids APPS study (*n* = 75).

	Mean (±SD) or *n* (%)
Parental age (y)	
25–34	25 (34%)
35–44	41 (57%)
45–54	6 (8%)
Parental gender (% woman)	68 (91%)
Parental educational attainment	
Some college or less	14 (19%)
College degree	28 (38%)
Graduate degree	32 (43%)
Annual household income^1^	
<$50,000	14 (19%)
$50,000–$99,999	17 (23%)
$100,000–$149,000	18 (24%)
>$150,000	24 (32%)
Child age (y)	
2–5	51 (68%)
6–11	24 (32%)
Child gender	
Boy	46 (61%)
Girl	28 (37%)
Nonbinary	1 (1%)
Child race/ethnicity	
White, non-Hispanic	50 (67%)
Black or African-American, non-Hispanic	7 (9%)
Hispanic, any race	7 (9%)
Multiracial	6 (8%)
Asian	4 (5%)
Perception of child’s screen use	
Too much	22 (31%)
Just right	46 (66%)
Not enough	2 (3%)
Child has own device	45 (62%)
Child uses social media (excluding YouTube)	4 (6%)
Parent uses a screen-time limiting app/parental control	13 (17%)

1 Two parents (3%) declined to report income.

Device usage data from device battery screenshots were submitted for 362 observation days across the 75 children; most (*n =* 256, 71%) observation days were weekdays (Table 2). YouTube was the most frequently used app, used at least once by 53% of the children, followed by YouTube Kids (23%), Netflix (23%), Prime Video (17%), and DisneyPlus (14%). Meanwhile, some type of game app was used at least once by 79% of children. The median estimated amount of total screen viewing time on mobile devices per child-day was 55 min (range: 0, 609), with large differences between weekday (median: 47.5 min) and weekend (median: 101.5 min) exposure.

**TABLE 2:** Average time spent on mobile devices, most frequent apps used, and exposure to marketing across *n =* 362^1^ observation days among *n =* 75 Massachusetts children aged 2–11 y old.

	Full sample	Parental educational attainment			Child gender		Child age	
		High school or less (*n =* 14 children, *n =* 69 child-days)	College graduate (*n =* 28 children, *n =* 130 child-days)	Graduate school (*n =* 32 children, *n =* 158 child-days)	Boy (*n =* 46 children, *n =* 222 child-days)	Girl^2^ (*n =* 29 children, *n =* 140 child-days)	2–5 y old (*n =* 51 children, *n =* 247 child-days)	6–11 y old (*n =* 24 children, *n =* 115 child-days)
Minutes per day spent on mobile devices [median (interquartile range)]	55 (12, 135)	95 (45, 204)	79 (23, 174)	45 (0,93)	54 (13, 144)	59 (11, 134)	53 (11, 155)	58 (15, 119)
Child viewed >120 min on mobile devices on a given day [*n* (%) of child-days]	102 (28%)	25 (36%)	45 (35%)	32 (20%)	64 (29%)	38 (27%)	74 (30%)	28 (24%)
Most frequent apps used [*n* (%) of child-days]								
YouTube	115 (32%)	41 (59%)	32 (25%)	42 (26%)	74 (33%)	41 (29%)	92 (37%)	23 (20%)
YouTube Kids	60 (17%)	10 (14%)	26 (20%)	24 (15%)	25 (11%)	35 (25%)	46 (19%)	14 (12%)
Individual game apps (e.g., Roblox)	178 (49%)	35 (51%)	72 (55%)	71 (44%)	106 (48%)	72 (51%)	109 (44%)	69 (60%)
Amazon Prime Video	22 (6%)	6 (8%)	10 (8%)	6 (4%)	16 (7%)	6 (4%)	18 (7%)	4 (3%)
Netflix	36 (10%)	0	18 (14%)	18 (11%)	23 (10%)	13 (9%)	18 (7%)	18 (16%)
DisneyPlus	27 (7%)	4 (6%)	14 (11%)	9 (6%)	17 (8%)	10 (7%)	17 (7%)	10 (9%)
Other^3^	11 (3%)	2 (3%)	3 (2%)	6 (4%)	6 (3%)	5 (4%)	9 (4%)	2 (2%)
Total food and beverage advertisements or branded product appearances seen per child-day on mobile devices [median (interquartile range)]	0 (0, 3)	1 (0,5)	0 (0,3)	0 (0,1)	0 (0,3)	0 (0,2)	0 (0,2)	0 (0,3)
Advertisements	0 (0, 0)	0 (0,0)	0 (0,0)	0 (0,0)	0 (0,0)	0 (0,0)	0 (0,0)	0 (0,0)
Brand appearances	0 (0, 3)	1 (0, 4)	0 (0, 2)	0 (0, 1)	0 (0, 2)	0 (0, 2)	0 (0, 2)	0 (0, 2)
Child viewed ≥2 food or beverage advertisements or brand appearances in given day [*n* (%) of child-days]	102 (28%)	32 (46%)	37 (28%)	33 (20%)	65 (29%)	37 (26%)	65 (26%)	37 (32%)

1 Range of observation days submitted per child: 4–5.

2 One nonbinary participant is included with girls.

3 Includes PBS Kids, Nick Jr, AppleTV, HBO Max, and Paramount Plus.

Although we estimated that children saw 2 or more food/beverage advertisements or brand appearances on 28% of observation days, and that 69% of the sample was exposed to any food/beverage advertisement during the study period, the distribution of exposure was highly variable. The median number of estimated food and beverage advertisements or brand appearances seen per day per child was 0, but ranged from 0 to 74. Children were only exposed on regular YouTube and Roblox (with a median exposure per each YouTube viewing of 2.0 ads, minimum 0, maximum 25, and a median exposure per each Roblox use of 0 ads, minimum 0, maximum 74), with no exposure on other apps. These results were similar to those found from the 29 subsample of participants whose parents viewed their child’s screen time alongside them and recorded advertising exposure (median exposure per day of 0; only exposures from YouTube). Examples of food and beverage advertisements and brand appearances seen in the sample are in Supplemental Figure 1. Across the 184 unique advertisements and branded product appearances seen in this study, the overwhelming majority of products advertised were less healthful foods and beverages, including sugary drinks (12%), sweets (36%), salty snacks (20%), and fast food restaurants (11%) (Supplemental Figure 2).

In validity analyses, parental estimates of screen time were moderately, but significantly, correlated with estimates derived from device battery screenshots [Spearman’s rho = 0.47, 95% confidence interval (CI): 0.28, 0.63, *P* < 0.001], but parents substantially underestimated their child’s screen time compared with device battery screenshot estimates (Table 3). Parents’ reporting of the proportion of observation days on which their child used various apps ranged in accuracy.

**TABLE 3:** Comparison of within-child averages of parent-reported screen time and characteristics of screen use with device battery screenshot-derived estimates, *n =* 75 children.

	Parent-reported estimate (median, min, max)	Device battery screenshot estimate (median, min, max)	Agreement [Spearman’s rho (95% confidence intervals)]^1^
*Mean daily screen time per child*	24 (0, 168)	66 (0, 395)	0.47 (0.28, 0.63)
*Use of mobile device apps(proportion ofobservation days per child withobserved use*)			
*YouTube*	0 (0, 1)	0.2 (0, 1)	0.84 (0.75, 0.89)
*YouTube Kids*	0 (0, 1)	0 (0, 1)	0.80 (0.69, 0.87)
*Netflix*	0 (0, 1)	0 (0, 1)	0.75 (0.63, 0.84)
*Amazon Prime Video*	0 (0, 0.4)	0 (0, 1)	0.42 (0.21, 0.59)
*DisneyPlus*	0 (0, 1)	0 (0, 1)	0.78 (0.67, 0.86)
*Game app (any kind)*	0.2 (0, 1)	0.7 (0, 1)	0.61 (0.45, 0.74)

1 All *P* values are <0.001.

In exploratory GEE models adjusting for weekend use, age and gender were not significantly associated with the odds of viewing 2 or more food or beverage ads per day. However, parental education was, with lower odds found for those with a graduate degree compared with those with less than a college degree (adjusted odds ratio = 0.26, 95% CI: 0.10, 0.70) and no difference between those with a college degree and those with less than a college degree. After additionally adjusting for total screen time per day, higher parental educational attainment was still associated with lower odds of seeing 2 or more ads per day (Table 4).

**TABLE 4:** Generalized estimating equation (GEE) model results testing associations between sociodemographic characteristics of children (*n =* 75) and likelihood per child-day of seeing 2+ food or beverage advertisements/branded product appearances, across *n =* 362 observation days [adjusted odds ratios (aORs), 95% confidence intervals].

	Minimally adjusted^1^	Fully adjusted^2^	Fully adjusted with inclusion of total screen time
Age category (y)			
2–5	(ref)	(ref)	(ref)
6–11	1.18 (0.49, 2.8)	1.63 (0.62, 4.25)	2.00 (0.78, 5.12)
Gender			
Girls^3^	(ref)	(ref)	(ref)
Boys	1.21 (0.51, 2.90)	1.02 (0.42, 2.50)	0.92 (0.38, 2.21)
Parental educational attainment			
High school or less	(ref)	(ref)	(ref)
College vs. high school or less	0.37 (0.13, 1.11)	0.33 (0.10, 1.06)	0.29 (0.10, 0.85)∗
Graduate school vs. high school or less	0.26 (0.10, 0.70)∗∗	0.22 (0.08, 0.64)∗∗	0.24 (0.08, 0.69)∗∗

∗∗∗ *P* < 0.001.

1 Model is adjusted for whether the observation day was a weekday or weekend day.

2 Model simultaneously includes age, gender, and parental education with weekday/weekend day status.

3 One participant identified as nonbinary; in the regression model, this participant was grouped with girls.

∗ *P* < 0.05

∗∗ *P* < 0.01

## Discussion

In this study, we estimated young children’s average daily exposure to food and beverage marketing on mobile devices over 5 d, finding it to be as low as 0 on a given day for most 2–11 y old children, but as high as 74 exposures per day for some, with exposure to marketing coming entirely from regular YouTube videos and various gaming apps. We also found higher estimated exposure to food and beverage marketing on devices for children of parents with lower educational attainment.

Our estimates of the amount of time young children spend on mobile devices are in line with previous studies; Rideout et al. [1] found in a 2020 national survey that 0–8-y olds were on average spending 55 min/d on digital devices, similar to our median estimate of 55 min. Although another study using similar methods to ours found mean screen time to be higher, at 115 min/d [42], we found a similar mean (95 min/d) and range (609 in this study compared with 632) [42], suggesting the distribution is likely similar; this prior study also found similar patterns in app usage, with YouTube being the most popular app. The high variability of daily screen time exposure in our sample also reflects previous investigations, which have found a large amount of heterogeneity both between and within individuals [35,47,48].

There was also substantial heterogeneity in estimated food and beverage marketing exposure. Although much of the sample was estimated to have been exposed to minimal amounts of food and beverage marketing on mobile devices, a fifth of the children were exposed to 2 or more instances of marketing per day. We further found a socioeconomic gradient, similarly to previous studies in non-United States contexts or with traditional television media [26,27], with children of parents with lower educational attainment exposed to more food and beverage marketing, regardless of any potential differences in screen time exposure. Focusing only on the sample average obscures that children who are already at risk for socioeconomic inequities in nutrition and health may be those who are being exposed more often to digital food and beverage marketing.

These socioeconomic differences in estimated exposure appear to be driven by the type of content viewed rather than differences in total screen time. Many children in this study used subscription streaming services for viewing videos—which, at the time of the study, did not show advertisements of any kind—effectively protecting them from advertisement exposure, if not from brand appearances. As more streaming services abandon subscription-only models and introduce advertising [49], children’s exposure is likely increasing, and socioeconomic disparities may be exacerbated given that services often charge higher subscription fees to allow for users to have fewer advertisements. Notably, almost all of the young children in our sample did not yet have social media apps that would require them to have a user account (for example, Instagram, Facebook, or TikTok) and thus were also protected from what may be one of the larger sources of exposure to food and beverage marketing for older youth [[31], [32], [33], [34]]. Children in our sample who most frequently used regular YouTube, however, were exposed to food and beverage advertising and brand appearances, echoing the findings of previous research that has specifically examined this app [29,30,50]. Although the YouTube Kids app does not allow for advertisements (although, notably, branded product appearances in videos are allowed), the regular YouTube app has no restrictions on advertising. The popular gaming app Roblox was also a key source of brand appearances, although not advertisements. The content-driven differences in food and beverage marketing exposure underscore the importance of considering not just how much time children spend on devices, but also whether they are engaging with content that is more likely to expose them to marketing.

Strengths of this study include the use of objective measures of screen time and advertising exposure, as well as the measurement of all screen time over several days, including both weekdays and weekends, which allowed us to capture participants’ heterogeneous patterns of device use. This exploratory study has several limitations, however, and our results should be interpreted with caution. Our convenience sample of 75 children is undoubtedly not representative; it is possible that both device usage and exposure to marketing would be different in a more representative sample. The small numbers of children with a race/ethnicity other than non-Hispanic White also precluded us from assessing potential racial/ethnic disparities in exposure, a key public health concern [44]. Similarly, income and education were highly collinear in our sample, precluding us from evaluating whether income had a different association with marketing exposure than education. Another limitation was that we were only able to measure children’s mobile device usage, which would have been in addition to their use or viewing of other devices (like televisions, computers, or videogame consoles). Children’s total daily exposure to food and beverage marketing, across all devices, would thus likely be much higher.

Although our measurement approach was adapted from previous approaches using objective sources of data on screen time and the amount of advertisements/brand appearances seen during children’s shows and games [29,30,42,43,50]—a substantial improvement over parental self-report measures—there is still risk for measurement error. Battery screenshots on shared devices, used by 38% of participants, present difficulties in teasing out which user accrued which time on which apps; we relied on parents’ reporting of how much of the shared device time was the child’s, but this may not be accurate. Although our measure of average marketing content per show or game also relied on objective measures, and is likely an accurate estimate of average exposure for a given child viewing the show or game, screen-based marketing is, of course, individually targeted, and thus our parent research assistants, using their child’s device, may not have seen exactly the same advertisements that another study child would have seen during a given video, although they would have seen the same brand appearances embedded within videos, which comprised most of the marketing exposure in this study. The parent research assistants may not have seen exactly the same stand-alone advertisements that their own child sees; although their use of their own child’s device meant that any cookies used for advertisement targeting would be on the device, it is possible that parents were still identifiable by advertisers as being adults through other, more immediate tracking techniques besides cookies, such as keyboard stroke patterns [11,36], although it is unclear how widely such tracking techniques are used and how much this would have affected estimates in this sample. Approaches that capture second-by-second images of individual users’ exposures, such as the Screenomics approach [51] or that ask participants to use screen-sharing software to share their device screens with researchers during shorter periods [38,41], would likely provide more accurate estimates of individuals’ exposure [38], although it is unclear whether their use might result in reactivity effects or otherwise impact users’ experiences in ways that would alter their regular device use patterns (that is, impacting device battery or speed). In this study, it was not feasible to use screen capturing software, because this would have necessitated a more intensive involvement of parents (because the children were too young to install and use it on their own) and because we were attempting to capture overall exposure across several days.

It is also unclear how advertising and brand appearance exposure on mobile devices impacts children’s dietary behavior. Although a robust literature has found substantial increases in caloric intake and intake of less healthful foods after exposure to television food and beverage advertising [23], the behavioral impact of digital marketing is less clear [52]. It is possible that digital marketing, with its individualized targeting and leveraging of social media influencers to promote products, has even stronger impacts on behavior than more broadly targeted television advertisements [13,53]. Future research should more thoroughly evaluate how digital food and beverage marketing influences eating behavior, and how different strategies (for example social media influencer promotions, product placement, social media challenges) may have different impacts.

In conclusion, young children in our sample had limited estimated exposure to food and beverage marketing on mobile devices on average. However, many children were at significantly higher risk for exposure due to using regular YouTube, which does not limit advertising, and children at higher risk for socioeconomic disadvantage were most exposed. Future research should continue to monitor young children’s exposure to digital food and beverage marketing and inequities in exposure using valid but feasible methods.

## Author contributions

The authors’ responsibilities were as follows – ELK, SNB: designed the research; RSM, JN: conducted the research; RSM, ELK: analyzed data; FF-M: consulted on the measurement design; ELK: wrote the paper; RSM, JN, FF-M, SNB: reviewed and edited the paper for important intellectual content; ELK: had primary responsibility for final content; and all authors: read and approved the final manuscript.

## Funding

This research was supported by Healthy Eating Research, a national program of the Robert Wood Johnson Foundation. The sponsor was not involved in the design, analysis, interpretation, or writing of this research.

## Data availability

Data described in the manuscript, code book, and analytic code will be made publicly and freely available without restriction at https://dataverse.harvard.edu/.

## Conflict of interest

The authors report no conflicts of interest.
