# Supplementary material for: Estimating Young Children’s Exposure to Food and Beverage Marketing on Mobile Devices
Source: Curr Dev Nutr. 2024 Nov 6;8(12):104505. doi: 10.1016/j.cdnut.2024.104505 (PMC11635717; doi:10.1016/j.cdnut.2024.104505)
Supplement: multimedia component 1 [file mmc1.docx]

**Supplementary Files for Kenney et al, “Estimating young children’s exposure to food and beverage marketing on mobile devices**”

**Supplementary Figure 1. Examples of advertisements and branded product appearances seen by children in the study sample.**


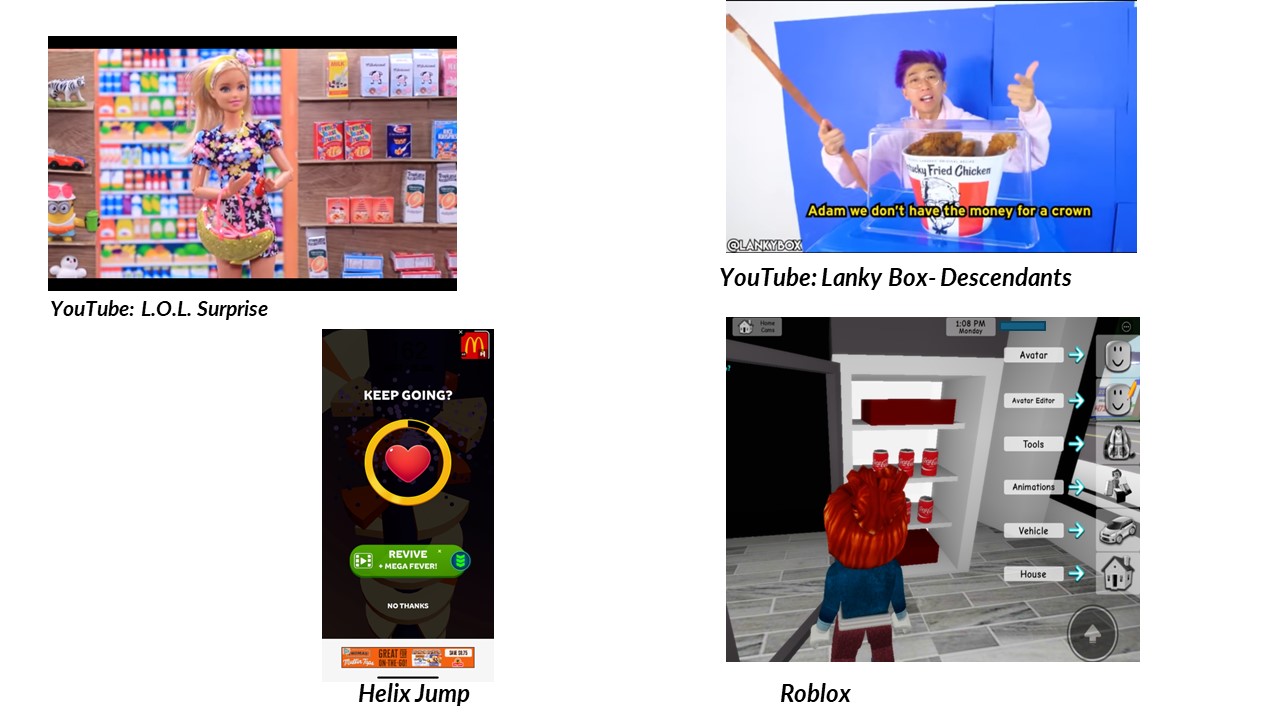


**Supplementary Figure 2. Percentage of 184 advertisements or branded product appearances promoting various types of foods or beverages.**


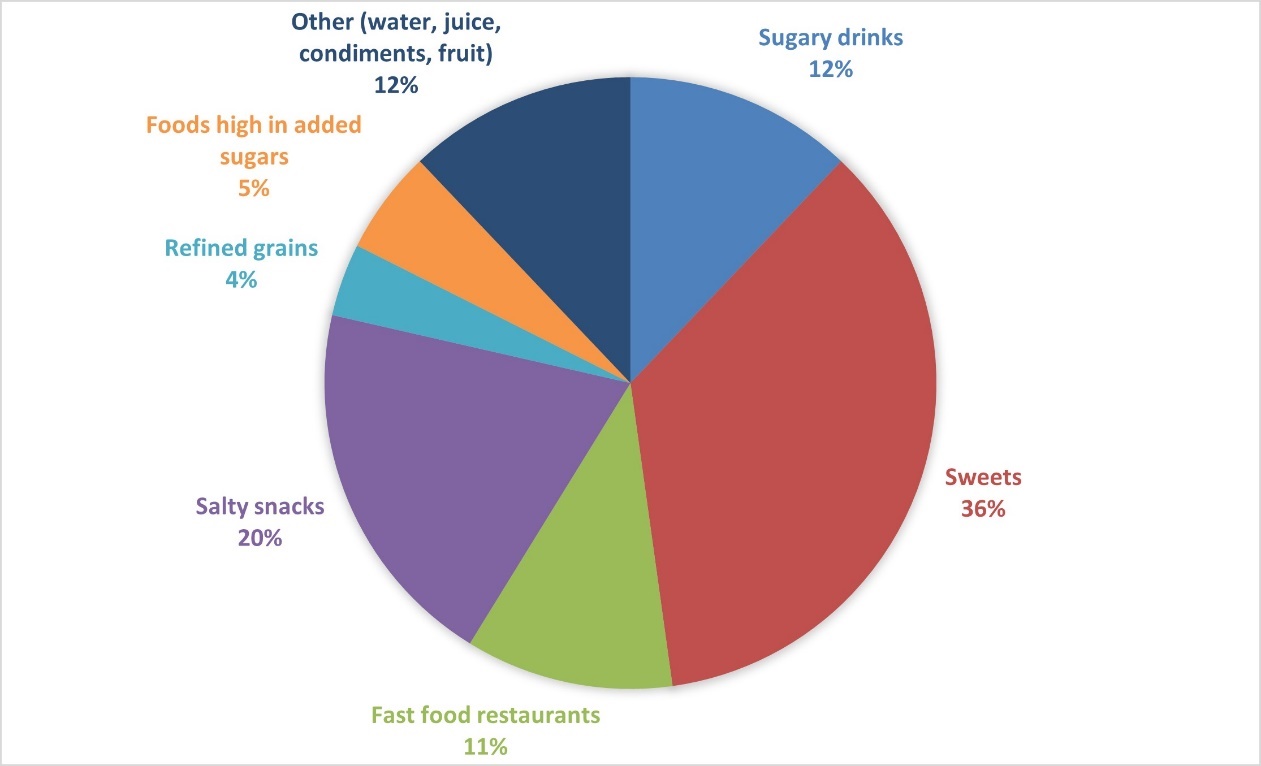


| **Example food codes:** |  |  |  |  |  |
| --- | --- | --- | --- | --- | --- |
| **Sugary drinks** | **Salty snacks** | **Foods high in added sugars** | **Sweets** | **Fast food restaurants** | **Refined grains** |
| Gatorade | Pizza | Cereals with added sugar >20% | Candy | Mc Donalds | Kellogg's Corn Flakes |
| Sprite | Chips |  | Bubble gum | Burger King | Hawaiian rolls |
| Red bull | Crackers |  | Ice cream | KFC |  |
|  | Pepperoni |  | Cookies |  |  |
|  | Pickles |  | Donuts |  |  |

**Supplementary Study Instrument 1: Initial parent survey**

Kids APPS Parent Survey

Start of Block: Default Question Block

Q1 Kids APPS Parent Survey

Q2 Please enter your last and first name (parent/guardian):

- Last: (1) ________________________________________________
- First (2) ________________________________________________

Q3 What is your age?

- 18-24 years old (1)
- 25-34 years old (2)
- 35-44 years old (3)
- 45-55 years old (4)
- > 55 years old (5)

Q4 What is your gender identity?

- Man (1)
- Woman (2)
- Non-binary / third gender (3)
- Prefer to self describe: (4) ________________________________________________

Q5 How would you best describe your race?

- Black or African American (1)
- White (2)
- Asian (3)
- American Indian or Alaska Native (4)
- Native Hawaiian or Other Pacific Islander (5)
- Other: (6) ________________________________________________

Q6 Are you of Hispanic/Latino/Spanish origin?

- Yes (1)
- No (2)

Q7 What language do you speak at home?

- English (1)
- Spanish (2)
- Other: (3) ________________________________________________

Q8 What is your highest educational degree obtained?

- Less than high school (1)
- High school diploma (2)
- Some college (3)
- College degree (4)
- Higher than college degree (5)
- Other: (6) ________________________________________________

Q9 How many children under the age of 18 live with you in your household?

________________________________________________________________

Q10 What is your household income per year?

- Less than $25,000 (1)
- $25,000 to $49,999 (2)
- $50,000 to $74,999 (3)
- $75,000 to $99,999 (4)
- $100,000 to $149,999 (5)
- More than $150,000 (6)
- Prefer not to say (7)

Q11 What is the zip code that you live in?

________________________________________________________________

End of Block: Default Question Block

Start of Block: Block 1

Q12 During the consenting process, we selected one child between 2-5 years of age to participate in this study. For the next set of questions, please respond for this child, who will be referred to as your STUDY CHILD.

Q13 What is the age of your study child?

________________________________________________________________

Q14 How does your study child identify in terms of gender?

- Boy (1)
- Girl (2)
- Non-binary (3)
- Prefer to self-describe (4) ________________________________________________

Q15 How would you best describe your study child's race?

- Black or African American (1)
- White (2)
- Asian (3)
- American Indian or Alaska Native (4)
- Native Hawaiian or Other Pacific Islander (5)
- Other: (6) ________________________________________________

Q16 Is your study child of Hispanic/Latino/Spanish origin?

- Yes (1)
- No (2)

Q17 How often does your study child attend child care?

- Every day (1)
- 2-4 days per week (2)
- Once a week (3)
- My child does not attend child care (4)

Q18 Please select what kind of child care your study child attends:

- Child care center (a larger number of children grouped by age in a non-residential location, multiple staff members) (1)
- Family child care home (a smaller number of children cared for in a private home or residence, 1-2 caregivers usually) (2)
- Preschool programs (offered for children ages 3-5 at school, childcare center, or other organization) (3)
- Family, friend, and neighbor care (4)
- My child does not attend child care (5)
- Other: (6) ________________________________________________

Q19 In what language does your study child primarily watch shows/videos or play games?

- English (1)
- Spanish (2)
- Other: (3) ________________________________________________

Q20 Thinking about how much time your study child spends with screen media, which of the following statements comes closest to your view?

- STUDY CHILD spends too MUCH time with screen media (1)
- STUDY CHILD spends too LITTLE time with screen media (2)
- STUDY CHILD spends the RIGHT amount of time with screen media (3)

Q21 Do you ever use any type of app for limiting your study child's screen time?

- Yes (1)
- If yes, what device or app do you use? (2) ________________________________________________
- No (3)
- Don't know (4)

Q22 For the following questions, approximately how old was your study child when they first did the following activities on a mobile device (for example, iPad, iPhone, Samsung Galaxy/ Android tablet, Amazon Fire, smartphone, iPod touch, etc)?

Q23 Watched movies or shows?

- < 2 years old (1)
- 2 to 3 years old (2)
- 3 to 5 years old (3)
- Never (4)

Q24 Watched videos, such as on YouTube?

- < 2 years old (1)
- 2 to 3 years old (2)
- 3 to 5 years old (3)
- Never (4)

Q25 Used apps for games?

- < 2 years old (1)
- 2 to 3 years old (2)
- 3 to 5 years old (3)
- Never (4)

| Page Break |  |
| --- | --- |

Q26 Please select the device or service that your study child uses most often to play games and watch videos (primary device):

- iPad (4)
- Samsung Galaxy Tablet or other Andriod tablet (5)
- Amazon Fire (not Kindle) (6)
- iPod Touch (7)
- Smartphone (8)
- Smart TV or TV with streaming device (Roku, Chromecast, AppleTV) (13)
- Cable or satellite TV (9)
- Laptop or desktop computer (10)
- Video game player (Xbox, PlayStation, Nintendo, Switch) (11)
- Other: (12) ________________________________________________

Q27 Is this primary device typically used at home or outside of your home?

- Typically used at home (1)
- Typically used outside of the home (2)
- About the same (3)
- Unsure (4)

Q28 My child uses a mobile device to play games or watch videos...

- At home more often than outside of our home (1)
- About the same amount at home as outside of our home (2)
- Outside of our home more often than at home (3)

| Page Break |  |
| --- | --- |

Q29 Please select the following subscription services that are on any of the devices that your study child uses:

- Netflix (1)
- Amazon Prime Video (2)
- Disney Plus (3)
- Hulu (4)
- YouTube or YouTube Kids (5)
- Nick Jr. (6)
- PBS Kids (7)
- Other: (8) ________________________________________________

| Page Break |  |
| --- | --- |

Q30 The following questions below are specifically about MOBILE DEVICES ONLY. For example, iPad, iPhone, Samsung Galaxy/ Android tablet, Amazon Fire, smartphone, iPod touch, etc.

Q31 How often does your study child watch movies, shows, videos, or play games on a mobile device ON WEEKDAYS (MONDAY THROUGH FRIDAY)?

- Never (1)
- Once a week (2)
- Several times a week (3)
- Once a day (4)
- Several times a day (5)

Q32 How often does your study child watch movies, shows, videos, or play games on a mobile device ON WEEKENDS (SATURDAYS AND SUNDAYS)?

- Never (1)
- Once on ONE weekend day (2)
- Once on BOTH weekend days (3)
- Several times on ONE weekend day (4)
- Several times on BOTH weekend days (5)

Q33 How often does your study child see ANY advertisements when using their mobile device?

- Never (1)
- Hardly ever (2)
- Sometimes (3)
- Often (4)
- Don't know (5)

Q34 How often does your study child see advertisements for drinks when using their mobile device? For example, juices, soda, sweet teas, milk etc.

- Never (1)
- Hardly ever (2)
- Sometimes (3)
- Often (4)
- Don't know (5)

Q35 For the next set of questions, please respond for YOURSELF, THE PARENT/GUARDIAN. When we ask about drinks, we are asking about non-alcoholic drinks only.


Examples of MOBILE DEVICES include iPad, iPhone, Samsung Galaxy/ Android tablet, Amazon Fire, smartphone, iPod touch, etc. 


Examples of SOCIAL MEDIA include Facebook, Instagram, Twitter, TikTok, etc.

Q36 How often do you see any advertisements while you are…

|  | Often (1) | Sometimes (2) | Rarely (3) | Never (4) |
| --- | --- | --- | --- | --- |
| Using a MOBILE DEVICE (8) |  |  |  |  |
| Watching TELEVISION (9) |  |  |  |  |
| Using SOCIAL MEDIA (10) |  |  |  |  |

Q37 How often do you see advertisements for SUGARY DRINKS (eg., soda, sweet teas, juice drinks, Gatorade/Powerade etc) while you are…

|  | Often (1) | Sometimes (2) | Rarely (3) | Never (4) |
| --- | --- | --- | --- | --- |
| Using a MOBILE DEVICE (8) |  |  |  |  |
| Watching TELEVISION (9) |  |  |  |  |
| Using SOCIAL MEDIA (10) |  |  |  |  |

Q38 How often do you see advertisements for UNSWEETENED DRINKS (eg., milk, water, seltzers, 100% juice, etc) while you are…

|  | Often (1) | Sometimes (2) | Rarely (3) | Never (4) |
| --- | --- | --- | --- | --- |
| Using a MOBILE DEVICE (8) |  |  |  |  |
| Watching TELEVISION (9) |  |  |  |  |
| Using SOCIAL MEDIA (10) |  |  |  |  |

Q39 Do you follow any non-alcoholic beverage brands on social media? For example, Coca-Cola, PepsiCo, Red Bull, Gatorade etc.

- No (1)
- Yes (2)
- If Yes, please enter brand(s): (3) ________________________________________________

Q40 Do you follow any food companies on social media? For example, Dunkin' Donuts, Taco Bell, McDonalds, Burger King, Kraft Foods, KFC, Wendy’s, etc.

- No (1)
- Yes (2)
- If Yes, please enter brand(s): (3) ________________________________________________

Q41 What is your study child's favorite beverage?

________________________________________________________________

Q42

 Do you serve your child any toddler milks or transitional formulas? If so, please list:

________________________________________________________________

**Supplementary Study Instrument 2: Daily Screen Time Survey**

Kids APPS Daily Screen Time Survey

Start of Block: Default Question Block

Q28 Please enter the following information:

Q1 What is your name (parent/guardian)?

- Last (1) ________________________________________________
- First (2) ________________________________________________

Q2 What day of the study are you filling out this survey for?

- Day 1 (1)
- Day 2 (2)
- Day 3 (3)
- Day 4 (4)
- Day 5 (5)

| Page Break |  |
| --- | --- |

Q3 Kids APPS Daily Screentime Survey

 Please respond to the following questions only for your child who was selected for this study (here referred to as your STUDY CHILD).

Q4 Please complete this survey once a day for all 5 study days—the same days for which you will be sending us the screenshots of device battery usage so we can measure how much time your child is spending on different apps.

Q5 Did your STUDY CHILD watch a movie, show, video or play games today on a mobile device?

- Yes (1)
- No (2)

Skip To: End of Survey If Did your STUDY CHILD watch a movie, show, video or play games today on a mobile device? = No

Q6 Which mobile device(s) did your child use that you will be sending screenshots from? Check all that apply.

- Smartphone (iPhone, Android, Galaxy) (1)
- iPad (2)
- Samsung Galaxy tablet (3)
- Android tablet (4)
- Amazon Fire (5)
- iPod Touch (6)
- Other device: (7) ________________________________________________

Q7 When your STUDY CHILD was using mobile devices today, did they:

- Watch videos/shows and play games (1)
- Watch videos/shows only (2)
- Play games only (3)

Skip To: End of Block If When your STUDY CHILD was using mobile devices today, did they: = Play games only

Q8 Below is a list of apps for viewing videos and shows on mobile devices. Please select the app(s) your child used today.

- YouTube (18)
- Youtube Kids (1)
- Netflix (2)
- Hulu (3)
- Amazon Prime Video (4)
- AppleTV (5)
- Disney Plus (6)
- Peacock (7)
- Nick, Jr. (8)
- Twitch (9)
- TikTok (10)
- HBO Max (11)
- PBS Kids (12)
- Paramount Plus (13)
- Philo (14)
- Frndly (15)
- SlingTV (16)
- Watched video from web browser (Chrome, Safari, etc.) (19)
- Other: (17) ________________________________________________

Carry Forward Selected Choices from "Below is a list of apps for viewing videos and shows on mobile devices. Please select the app(s) your child used today. "

| 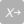 |
| --- |

Q9 Please list the names of all shows or videos watched today on the apps you selected:

- YouTube (1) ________________________________________________
- Youtube Kids (2) ________________________________________________
- Netflix (3) ________________________________________________
- Hulu (4) ________________________________________________
- Amazon Prime Video (5) ________________________________________________
- AppleTV (6) ________________________________________________
- Disney Plus (7) ________________________________________________
- Peacock (8) ________________________________________________
- Nick, Jr. (9) ________________________________________________
- Twitch (10) ________________________________________________
- TikTok (11) ________________________________________________
- HBO Max (12) ________________________________________________
- PBS Kids (13) ________________________________________________
- Paramount Plus (14) ________________________________________________
- Philo (15) ________________________________________________
- Frndly (16) ________________________________________________
- SlingTV (17) ________________________________________________
- Watched video from web browser (Chrome, Safari, etc.) (18) ________________________________________________
- Other: (19) ________________________________________________

Display This Question:

If Below is a list of apps for viewing videos and shows on mobile devices. Please select the app(s)... = YouTube

Q10 How long did your child watch YouTube?

- 0 - 15 minutes (4)
- 15 - 30 minutes (5)
- 30 minutes - 1 hour (6)
- 1 - 1.5 hour (7)
- 1.5 - 2 hours (8)
- More than 2 hours (9)

Display This Question:

If Below is a list of apps for viewing videos and shows on mobile devices. Please select the app(s)... = Youtube Kids

Q11 How long did your child watch YouTube Kids?

- 0 - 15 minutes (4)
- 15 - 30 minutes (5)
- 30 minutes - 1 hour (6)
- 1 - 1.5 hour (7)
- 1.5 - 2 hours (8)
- More than 2 hours (9)

Display This Question:

If Below is a list of apps for viewing videos and shows on mobile devices. Please select the app(s)... = Netflix

Q12 How long did your child watch Netflix?

- 0 - 15 minutes (4)
- 15 - 30 minutes (5)
- 30 minutes - 1 hour (6)
- 1 - 1.5 hour (7)
- 1.5 - 2 hours (8)
- More than 2 hours (9)

Display This Question:

If Below is a list of apps for viewing videos and shows on mobile devices. Please select the app(s)... = Hulu

Q13 How long did your child watch Hulu?

- 0 - 15 minutes (4)
- 15 - 30 minutes (5)
- 30 minutes - 1 hour (6)
- 1 - 1.5 hour (7)
- 1.5 - 2 hours (8)
- More than 2 hours (9)

Display This Question:

If Below is a list of apps for viewing videos and shows on mobile devices. Please select the app(s)... = Amazon Prime Video

Q14 How long did your child watch Amazon Prime?

- 0 - 15 minutes (4)
- 15 - 30 minutes (5)
- 30 minutes - 1 hour (6)
- 1 - 1.5 hour (7)
- 1.5 - 2 hours (8)
- More than 2 hours (9)

Display This Question:

If Below is a list of apps for viewing videos and shows on mobile devices. Please select the app(s)... = AppleTV

Q15 How long did your child watch AppleTV?

- 0 - 15 minutes (4)
- 15 - 30 minutes (5)
- 30 minutes - 1 hour (6)
- 1 - 1.5 hour (7)
- 1.5 - 2 hours (8)
- More than 2 hours (9)

Display This Question:

If Below is a list of apps for viewing videos and shows on mobile devices. Please select the app(s)... = Disney Plus

Q16 How long did your child watch Disney Plus?

- 0 - 15 minutes (4)
- 15 - 30 minutes (5)
- 30 minutes - 1 hour (6)
- 1 - 1.5 hour (7)
- 1.5 - 2 hours (8)
- More than 2 hours (9)

Display This Question:

If Below is a list of apps for viewing videos and shows on mobile devices. Please select the app(s)... = Peacock

Q17 How long did your child watch Peacock?

- 0 - 15 minutes (4)
- 15 - 30 minutes (5)
- 30 minutes - 1 hour (6)
- 1 - 1.5 hour (7)
- 1.5 - 2 hours (8)
- More than 2 hours (9)

Display This Question:

If Below is a list of apps for viewing videos and shows on mobile devices. Please select the app(s)... = Nick, Jr.

Q18 How long did your child watch Nick, Jr.?

- 0 - 15 minutes (4)
- 15 - 30 minutes (5)
- 30 minutes - 1 hour (6)
- 1 - 1.5 hour (7)
- 1.5 - 2 hours (8)
- More than 2 hours (9)

Display This Question:

If Below is a list of apps for viewing videos and shows on mobile devices. Please select the app(s)... = Twitch

Q19 How long did your child watch Twitch?

- 0 - 15 minutes (4)
- 15 - 30 minutes (5)
- 30 minutes - 1 hour (6)
- 1 - 1.5 hour (7)
- 1.5 - 2 hours (8)
- More than 2 hours (9)

Display This Question:

If Below is a list of apps for viewing videos and shows on mobile devices. Please select the app(s)... = TikTok

Q20 How long did your child watch TikTok?

- 0 - 15 minutes (4)
- 15 - 30 minutes (5)
- 30 minutes - 1 hour (6)
- 1 - 1.5 hour (7)
- 1.5 - 2 hours (8)
- More than 2 hours (9)

Display This Question:

If Below is a list of apps for viewing videos and shows on mobile devices. Please select the app(s)... = HBO Max

Q21 How long did your child watch HBO Max?

- 0 - 15 minutes (4)
- 15 - 30 minutes (5)
- 30 minutes - 1 hour (6)
- 1 - 1.5 hour (7)
- 1.5 - 2 hours (8)
- More than 2 hours (9)

Display This Question:

If Below is a list of apps for viewing videos and shows on mobile devices. Please select the app(s)... = PBS Kids

Q22 How long did your child watch PBS Kids?

- 0 - 15 minutes (4)
- 15 - 30 minutes (5)
- 30 minutes - 1 hour (6)
- 1 - 1.5 hour (7)
- 1.5 - 2 hours (8)
- More than 2 hours (9)

Display This Question:

If Below is a list of apps for viewing videos and shows on mobile devices. Please select the app(s)... = Paramount Plus

Q23 How long did your child watch Paramount Plus?

- 0 - 15 minutes (4)
- 15 - 30 minutes (5)
- 30 minutes - 1 hour (6)
- 1 - 1.5 hour (7)
- 1.5 - 2 hours (8)
- More than 2 hours (9)

Display This Question:

If Below is a list of apps for viewing videos and shows on mobile devices. Please select the app(s)... = Philo

Q24 How long did your child watch Philo?

- 0 - 15 minutes (4)
- 15 - 30 minutes (5)
- 30 minutes - 1 hour (6)
- 1 - 1.5 hour (7)
- 1.5 - 2 hours (8)
- More than 2 hours (9)

Display This Question:

If Below is a list of apps for viewing videos and shows on mobile devices. Please select the app(s)... = Frndly

Q25 How long did your child watch Frndly?

- 0 - 15 minutes (4)
- 15 - 30 minutes (5)
- 30 minutes - 1 hour (6)
- 1 - 1.5 hour (7)
- 1.5 - 2 hours (8)
- More than 2 hours (9)

Display This Question:

If Below is a list of apps for viewing videos and shows on mobile devices. Please select the app(s)... = SlingTV

Q50 How long did your child watch SlingTV?

- 0 - 15 minutes (4)
- 15 - 30 minutes (5)
- 30 minutes - 1 hour (6)
- 1 - 1.5 hour (7)
- 1.5 - 2 hours (8)
- More than 2 hours (9)

Display This Question:

If Below is a list of apps for viewing videos and shows on mobile devices. Please select the app(s)... = Watched video from web browser (Chrome, Safari, etc.)

Q49 How long did your child watch videos on the web browser?

- 0 - 15 minutes (1)
- 15 - 30 minutes (2)
- 30 minutes - 1 hour (3)
- 1 - 1.5 hour (4)
- 1.5 - 2 hours (5)
- More than 2 hours (6)

Display This Question:

If Below is a list of apps for viewing videos and shows on mobile devices. Please select the app(s)... = Amazon Prime Video

Q53 Please select the service you subscribe to within this app.

- Amazon Prime Video - free version (1)
- Amazon Prime Video - Yearly ($119/year) (2)
- Amazon Prime Video - Monthly ($12.99/month) (3)

Display This Question:

If Below is a list of apps for viewing videos and shows on mobile devices. Please select the app(s)... = Hulu

Q54 Please select the service you subscribe to within this app.

- Hulu ($6.99/month) (1)
- Hulu, No Ads ($12.99/month) (2)

Display This Question:

If Below is a list of apps for viewing videos and shows on mobile devices. Please select the app(s)... = Disney Plus

Q55 Please select the service you subscribe to within this app.

- Disney+, Monthly ($7.99/month) (1)
- Disney+, Yearly ($79.99/year) (2)

Display This Question:

If Below is a list of apps for viewing videos and shows on mobile devices. Please select the app(s)... = HBO Max

Q56 Please select the service you subscribe to within this app.

- HBO Max, with ads ($9.99/month) (1)
- HBO Max, ad-free ($14.99/month) (2)

Display This Question:

If Below is a list of apps for viewing videos and shows on mobile devices. Please select the app(s)... = SlingTV

Q57 Please select the service you subscribe to within this app.

- Sling TV, Sling Orange ($35/month) (1)
- Sling TV, Sling Blue ($35/month) (2)
- Sling TV, both Sling Orange/Blue ($50/month) (4)

Display This Question:

If Below is a list of apps for viewing videos and shows on mobile devices. Please select the app(s)... = Peacock

Q58 Please select the service you subscribe to within this app.

- Peacock, Premium ($4.99/month) (1)
- Peacock, Premium Plus ($9.99/month) (2)

Display This Question:

If Below is a list of apps for viewing videos and shows on mobile devices. Please select the app(s)... = Paramount Plus

Q59 Please select the service you subscribe to within this app.

- Peacock, Premium ($4.99/month)Paramount Plus, 1 year, limited commercials ($49.99/year) (1)
- Paramount Plus, 1 month, limited commercials ($4.99/month) (2)
- Paramount Plus, 1 month, no commercials ($9.99/month) (5)

End of Block: Default Question Block

Start of Block: Block 1

Display This Question:

If When your STUDY CHILD was using mobile devices today, did they: = Watch videos/shows and play games

Or When your STUDY CHILD was using mobile devices today, did they: = Play games only

Q48 What game did your child play? For how long?

- What game? (4) ________________________________________________
- How long did they play? (5) ________________________________________________

Display This Question:

If Did your STUDY CHILD watch a movie, show, video or play games today on a mobile device? = No

Q47 If your child did not use a mobile device on this study day, please email or text apps.harvard@gmail.com or 617-299-0837 the message: "My child did not use a mobile device on study day ___." Please include your full name and the study day. Thank you!

Display This Question:

If Did your STUDY CHILD watch a movie, show, video or play games today on a mobile device? = Yes

Q46 Please remember to email or text screenshots (or a photo taken) of your child's device usage to apps.harvard@gmail.com or 617-299-0837. Thank you!

Q27 Thank you for completing this survey. To review your entries, please select the back arrow, or select the forward arrow to submit your responses.
